# Supplementary material for: ENO2 Promotes Colorectal Cancer Metastasis by Interacting with the LncRNA CYTOR and Activating YAP1-Induced EMT
Source: Cells. 2022 Aug 1;11(15):2363. doi: 10.3390/cells11152363 (PMC9367517; doi:10.3390/cells11152363)
Supplement: Supplementary file 1 [file cells-11-02363-s001.zip › cells-1821456-supplementary/Supp.-PUB/Supporting informationl-FigureS1.pdf]

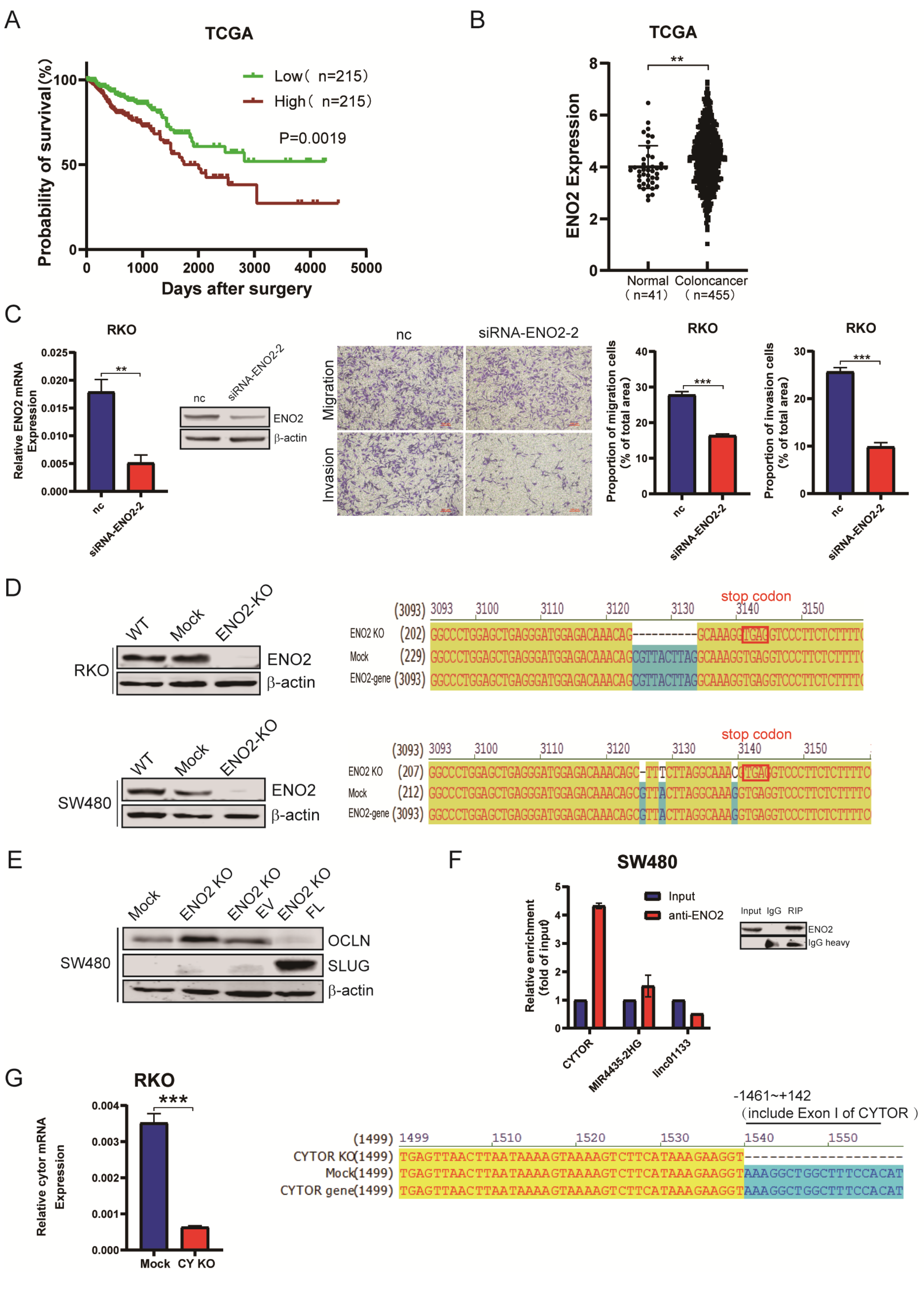

A
TCGA
Low（ n=215）
High（ n=215）
P=0.0019
Probability of survival(%)
Days after surgery
B
TCGA
ENO2 Expression
Normal （n=41）
Coloncancer （n=455）
C
RKO
Relative ENO2 mRNA Expression
nc
siRNA-ENO2-2
ENO2
β-actin
Migration
Invasion
Proportion of migration cells (% of total area)
Proportion of invasion cells (% of total area)
D
WT
Mock
ENO2-KO
RKO
SW480
ENO2
β-actin
stop codon
ENO2 KO
Mock
ENO2-gene
E
Mock
ENO2 KO
ENO2 KO EV
ENO2 KO FL
SW480
OCLN
SLUG
β-actin
F
SW480
Input
anti-ENO2
Relative enrichment (fold of input)
CYTOR
MIR4435-2HG
linc01133
Input IgG RIP
ENO2
IgG heavy
G
RKO
Relative cytor mRNA Expression
Mock
CY KO
-1461~+142
（include Exon I of CYTOR ）
CYTOR KO(1499) TGAGTTAACTTAATAAAAGTAAAAGTCTTCATAAAGAAGGT
Mock(1499) TGAGTTAACTTAATAAAAGTAAAAGTCTTCATAAAGAAGGTAAAGGCTGGCTTTCCACAT
CYTOR gene(1499) TGAGTTAACTTAATAAAAGTAAAAGTCTTCATAAAGAAGGTAAAGGCTGGCTTTCCACAT

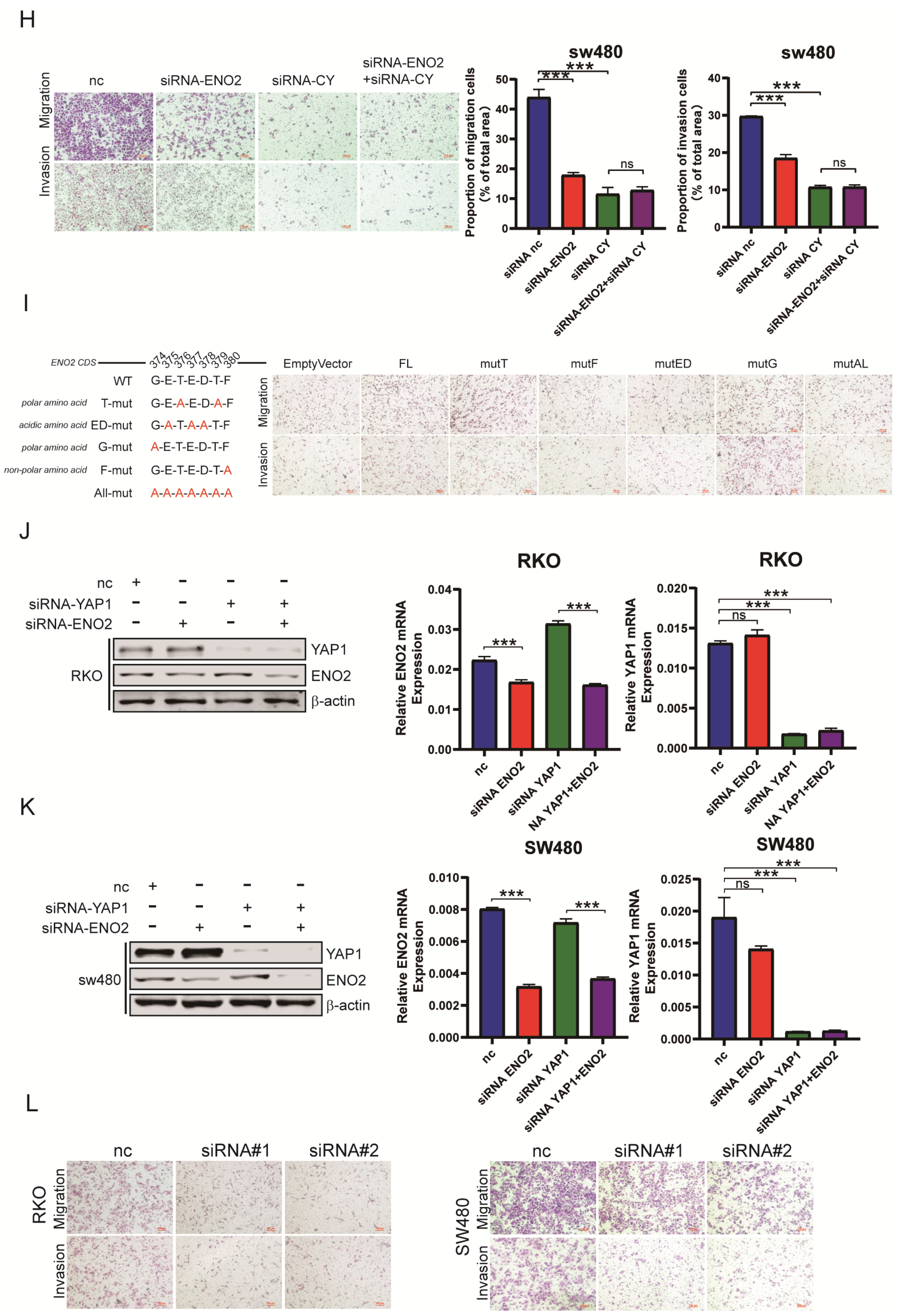
H
nc
siRNA-ENO2
siRNA-CY
siRNA-ENO2 +siRNA-CY
Migration
Invasion
sw480
Proportion of migration cells (% of total area)
Proportion of invasion cells (% of total area)
***
ns
siRNA nc
siRNA-ENO2
siRNA CY
siRNA-ENO2+siRNA CY
I
ENO2 CDS
374 375 376 377 378 379 380
WT G-E-T-E-D-T-F
polar amino acid T-mut G-E-A-E-D-A-F
acidic amino acid ED-mut G-A-T-A-A-T-F
polar amino acid G-mut A-E-T-E-D-T-F
non-polar amino acid F-mut G-E-T-E-D-T-A
All-mut A-A-A-A-A-A-A
EmptyVector
FL
mutT
mutF
mutED
mutG
mutAL
J
nc
siRNA-YAP1
siRNA-ENO2
RKO
YAP1
ENO2
β-actin
Relative ENO2 mRNA Expression
Relative YAP1 mRNA Expression
siRNA ENO2
siRNA YAP1
NA YAP1+ENO2
K
sw480
SW480
siRNA YAP1+ENO2
L
siRNA#1
siRNA#2

Figure S1 ENO2 promotes colorectal cancer metastasis by interacting with CYTOR and activating YAP1-induced EMT

A: Prognostic role of ENO2 expression in TCGA cohort.

B: ENO2 expression in CRC and normal colon tissues.

C: Identification of ENO2 knockdown by another siRNA-ENO2 on RKO migration and invasion. Shown from left to right are mRNA as measured by qPCR, protein levels as measured by western blot, representative transwell images and statistical results from the transwell migration and invasion assays.

D: Identification of RKO ENO2 KO and SW480 ENO2 KO. Left: The expression of ENO2 in wild type (WT), mock control (Mock) and ENO2KO cells were tested by WB. Right: Alignment of ENO2KO sequencing results. Red box indicates stop codon.

E:The expression of EMT-related genes after reintroducing ENO2 into ENO2-silenced SW480 cells.

F: RIP analysis of endogenous ENO2 binding to RNA in SW480 cells.

G: Identification of CYTOR KO in RKO. Left: The expression of CYTOR in mock control (Mock) and CYTOR-KO cells were tested by qPCR. Right: Alignment of CYTOR-KO sequencing results. The region of promoter and Exon I of CYTOR was deleted.

H: Migration and invasion of SW480 cells with CYTOR and ENO2 knockdown.

I: Schematic representation and functions of single point mutations to amino acid residues 374–380 of ENO2.

J-K: Characterization of simultaneous knockdown of ENO2 and YAP1 in RKO and SW480 cells. Protein and RNA expression are reflected by western blot and qPCR results.

L: The migration and invasion of RKO cells with YAP1 knockdown(left)and SW480 YAP1 knockdown(right). Data are shown as the mean ± SD., ***P*<0.01, ****P*<0.001.
