# Supplementary material for: ENO2 Promotes Colorectal Cancer Metastasis by Interacting with the LncRNA CYTOR and Activating YAP1-Induced EMT
Source: Cells. 2022 Aug 1;11(15):2363. doi: 10.3390/cells11152363 (PMC9367517; doi:10.3390/cells11152363)
Supplement: Supplementary file 1 [file cells-11-02363-s001.zip › cells-1821456-supplementary/Supp.-PUB/Supporting_information-TableS1.pdf]

TableS1 Key resources table

REAGENT or RESOURCE	SOURCE	IDENTIFIER
Antibodies		
Mouse monoclonal anti-ENO2	Santa Cruz	sc-376375
Rabbit monoclonal anti-ENO2	CST	Cat#6516S
Mouse monoclonal anti-GAPDH	Multi-sciences	Cat#A85370844
Monoclonal ANTI-FLAG® M2 antibody	Sigma	F1804-50UG
Mouse monoclonal anti- Actin	Multi-sciences	Cat#70-ab008
Rabbit monoclonal anti-YAP1	CST	Cat#3724S
Rabbit monoclonal anti-p-YAP1	CST	Cat#13008
Rabbit monoclonal anti-E-cadherin	CST	Cat#3195S
Rabbit monoclonal anti-OCLN	Abcam	ab216327
Rabbit monoclonal anti-SNAI	CST	Cat#3879S
Rabbit monoclonal anti-SLUG	Abcam	ab27568
Mouse monoclonal anti-LATS1	Santa Cruz	sc-398560
Rabbit monoclonal anti-LATS1	CST	Cat#3477T
Rabbit monoclonal anti-LATS2	CST	Cat#5888T
Rabbit IgG(H+L)	CST	Cat#3900
Mouse IgG(H+L)	Santa Cruz	sc-2025
Alexa® Fluor 488 Donkey anti-Mouse IgG (H+L)	ThermoFisher	Cat#A-21202
Alexa® Fluor 546 Goat anti-Rabbit IgG (H+L)	ThermoFisher	Cat#A-11010
IRDye® 800CW Goat-anti-Rabbit Antibody	LI-COR	Cat#926-32211
IRDye® 680CW Goat-anti-Mouse Antibody	LI-COR	Cat#926-68070
Bacterial		
DH5a	Vazyme	C502-02
Biological Samples		
Patient samples	Run RunShaw HospitalZhejiang University	
Chemicals, Peptides, and Recombinant Proteins		
D-luciferin	Goldbio	Cat#LUCNA (Luciferin, Potassium Salt)
G 418 disulfate salt	Sigma	G 5013
GenMute siRNA Transfection Reagent	SignaGen	Cat # SL100568
Human Plasma Fibronectin Purified Protein	Millipore	FC010-1MG
Lipofectamine2000	Invitrogen	Cat#11668027
LipoD293	SignaGen	Cat # SL100668
Matrigel	BD Biosciences	Cat #356234
Puromycin	Sigma	Cat#P8833-10MG
Trizol	Invitrogen	Cat#15596018
Critical Commercial Assays		
Anti-FLAG® M2 Magnetic Beads	Sigma	Cat#M8823
BCA protein assay	ThermoFisher	Cat#23225
ClonExpress-II One Step Cloning Kit	Vazyme	C112

Seahorse XF Glycolytic Rate Assay Kit	Agilent	103344-100
Mut Express II Fast Mutagenesis Kit V2	Vazyme	C214
Protein A/G PLUS-Agarose	Santa Cruz	sc-2003
Oligonucleotides		
Primers for QPCR, see below	hzyKang	Table S2
siRNA ENO2-1	GenePharma	
siRNA ENO2-2	GenePharma	
siRNA CYTOR	GenePharma	
siRNA si-PKM2	GenePharma	
siRNA YAP1-1	GenePharma	
siRNA YAP1-2	GenePharma	
siRNA Negative Control	GenePharma	
