# Supplementary material for: ENO2 Promotes Colorectal Cancer Metastasis by Interacting with the LncRNA CYTOR and Activating YAP1-Induced EMT
Source: Cells. 2022 Aug 1;11(15):2363. doi: 10.3390/cells11152363 (PMC9367517; doi:10.3390/cells11152363)
Supplement: Supplementary file 1 [file cells-11-02363-s001.zip › cells-1821456-supplementary/Supp.-PUB/Supporting_information-TableS2.pdf]

TableS2 Nucleotide sequence

Primers For qPCR

ENO2	CGTTACTTAGGCAAAGGTGTCC	CTCCAGCATCAGGTTGTCCAGT
ACTIN	CATGTACGTTGCTATCCAGGC	CTCCTTAATGTCACGCACGAT
CYTOR	GACCCAGCAACCATCTCCTG	GGGGGCTGAGTCGTGATTTT
PKM2	TGTCGAAGCCCCATAGTGAA	GGGTGGTGAATCAATGTCCA
YAP1	TAGCCCTGCGTAGCCAGTTA	CATGCTTAGTCCACTGTCTGT
MIR4435-2HG	GACATTCCAGACAAGCGGTG	GAAAAGATGCTGGTGACTGC
LINC01133	CTAATCTCACCACAGCCTGG	GCCTGGATTACCAATGAGGA
CDH1	CGAGAGCTACACGTTCACGG	GGTGTCGAGGGAAAAATAGG
CDH2	GCCAACCTTAACTGAGGAGT	GCAAGTTGATTGGAGGGATG
SLUG	ACCTTGTGTTTGCAAGATCTGC	TGCAAATGCTCTGTTGCAGTGAG
OCLN	ACAAGCGGTTTTATCCAGAGTC	GTCATCCACAGGCGAAGTTAAT

ShRNA oligos

shENO2-1	CGTTCTGAACGTCTGGCTAAA	TTTAGCCAGACGTTCAGAACG
shENO2-2	GCCGGACATAACTTCCGTAAT	ATTACGGAAGTTATGTCCGGC
shENO2-3	CAAACAGCGTTACTTAGGCAA	TTGCCTAAGTAACGCTGTTTG

SiRNA sequences

si-ENO2-1	CGUUCUGAACGUCUGGCUAAATT	UUUAGCCAGACGUUCAGAACGTT
si-ENO2-2	CAAGGGAGUCAUCAAGGACAATT	UUGUCCUUGAUGACUCCCUUGTT
si-CYTOR	CAGUCUCUAUGUGUCUUAATT	UUAAGACACAUAGAGACUGTT
si-PKM2	GUGGAUGAUGGGCUUAUUUCTT	GAAAUAAGCCCAUCAUCCACGTT
si-YAP1-1	GCCACCAAGCUAGAUAAAGAATT	UUCUUUAUCUAGCUUGGUGGCTT
si-YAP1-2	CAGGUGAUACUAUCAACCAAATT	UUUGGUUGAUAGUAUCACCUGTT

Overexpress and single point mutants primers

ENO2-FL-Flag	gatgacgatgacaaggatatcATGTCCA TAGAGAAGATCTGGGCC	atccttcgcggccgcggatccTCACAG CACACTGGGATTACGG
ENO2 1-304aa	gatgacgatgacaaggatatcATGTCCA TAGAGAAGATCTGGGCC	atccttcgcggccgcggatccTCACCA GGCAGCCCAATCA
ENO2 305-434aa	gatgacgatgacaaggatatcATGTCCA AGTTCACAGCCAATGT	atccttcgcggccgcggatccTCACAG CACACTGGGATTACGG
ENO2 1-369aa	gatgacgatgacaaggatatcATGTCCA TAGAGAAGATCTGGGCC	atccttcgcggccgcggatccTCACAC CATGACCCCCCA
ENO2 367-434aa	gatgacgatgacaaggatatcATGGTGA GTCATCGCTCAGGA	atccttcgcggccgcggatccTCACAG CACACTGGGATTACGG
ENO2 1-380aa	gatgacgatgacaaggatatcATGTCCA TAGAGAAGATCTGGGCC	atccttcgcggccgcggatccTCAGAA TGTGTCCTCAGTCTCTCCT
ENO2-SBD-mut	atggtggctgctgctgctGGAGAGACT GAGGACACATTC	agcagcagcagcCACCATGACCC CCCAGCCATT
ENO2-T-mut	agaggctgaggacgctttcATTGCTGA CCTGGTGGTGGG	aagcgtcctcagcctctccTGAGCGAT GACTCACCATGACC
ENO2-F-mut	agagactgaggacacagctATTGCTGA CCTGGTGGTGGG	ctgtgtcctcagtctctccTGAGCGAT GACTCACCATGACC
ENO2-ED-mut	agctactgctgctacattcATTGCTGAC	atgtagcagcagtagctccTGAGCGA

	CTGGTGGTGGG	TGACTCACCATGACC
ENO2-G-mut	tgagactgaggacacattcATTGCTGA CCTGGTGGTGGG	atgtgtcctcagtctcagcTGAGCGAT GACTCACCATGACC
ENO2-AL-mut	tgctgctgctgctgctgctATTGCTGAC CTGGTGGTGGG	cagcagcagcagcagcagcTGAGCG ATGACTCACCATGAC
