# Supplementary material for: ENO2 Promotes Colorectal Cancer Metastasis by Interacting with the LncRNA CYTOR and Activating YAP1-Induced EMT
Source: Cells. 2022 Aug 1;11(15):2363. doi: 10.3390/cells11152363 (PMC9367517; doi:10.3390/cells11152363)
Supplement: Supplementary file 1 [file cells-11-02363-s001.zip › cells-1821456-supplementary/Supp.-PUB/Supporting_information-TableS3.pdf]

TableS3 Relationship between ENO2 expression and clinical pathological characteristic of CRC patients

Characteristics	Number (%)	ENO2 expression			χ^2	*P* value
		High(%)	Middle(%)	Low(%)		
Age						
≤60	79(42.9)	27(14.7)	30(16.3)	22(11.9)	3.75	0.153
＞60	105(57.1)	26(14.1)	36(19.6)	43(23.4)		
Gender						
Male	103(56.5)	35(19)	37(20.1)	31(16.8)	3.988	0.136
Female	81(43.5)	18(9.8)	29(15.8)	34(18.5)		
Tumor site★						
Colon	101(54.9)	32(17.4)	40(21.7)	29(15.8)	3.494	0.174
Rectal	81(44)	21(11.4)	26(14.1)	34(18.5)		
Tumor size★						
≤5 cm	112(60.9)	31(16.8)	39(21.2)	42(22.8)	0.711	0.701
＞5 cm	64(34.8)	19(10.3)	25(13.6)	20(10.9)		
Histological type						
tubular adenocarcinoma	131(71.2)	37(20.1)	49(26.6)	45(24.5)	0.471	0.79
other type	53(28.8)	16(8.7)	17(9.2)	20(10.9)		
Degree of differentiation★						
High/moderate	136(73.9)	35(19)	48(26)	53(28.8.)	4.411	0.11
Poor/undifferentiated	47(25.5)	18(9.8)	18(9.8)	11(5.9)		
Gross type						
Ulcerative type	102(55.4)	34(18.5)	38(20.7)	30(16.3)	4.019	0.134
Other type	82(44.6)	19(10.3)	28(15.2)	35(19)		
Lymph node invasion						
N0	102(55.4)	29(15.8)	36(19.6)	37(20.1)	0.514	0.773
N1	77(41.8)	24(13)	29(15.8)	24(13)		
Unknown	5(2.7)					
Vascular invasion						
N0	121(65.8)	33(17.9)	47(25.5)	41(22.3)	0.199	0.905
N1	51(27.7)	15(8.2)	18(9.8)	18(9.8)		
Unknown	12(6.5)					
TNM stage						
I/II	173(94.0)	50(27.2)	64(34.8)	59(32.1)	2.253	0.324
III/IV	11(6.0)	3(1.6)	2(1.1)	6(3.3)		
Survival						
≤5 years	56(30.4)	21(11.4)	19(10.3)	16(8.7)	8.312	0.016
＞5 years	128(69.6)	32(17.4)	47(25.6)	49(26.6)		

★Patients with missing or inconclusive statistical information were excluded.

Statistical differences were calculated using the Chi-Square test
